# Current Status of Malaria Control and Elimination in Africa: Epidemiology, Diagnosis, Treatment, Progress and Challenges

**DOI:** 10.1007/s44197-024-00228-2

**Published:** 2024-04-24

**Authors:** Jiahuan Li, Haragakiza Jean Docile, David Fisher, Khrystyna Pronyuk, Lei Zhao

**Affiliations:** 1 Department of Infectious Diseases, Union Hospital, Tongji Medical College, Huazhong University of Science and Technology, Wuhan, People’s Republic of China; 2 Department of Medical Biosciences, Faculty of Natural Sciences, University of The Western Cape, Cape Town, South Africa; 3 Department of Infectious Diseases, O. Bogomolets National Medical University, Kyiv, Ukraine

**Keywords:** Malaria, Epidemiology, Diagnosis, Prevention, Treatment, Africa

## Abstract

The African continent carries the greatest malaria burden in the world. Falciparum malaria especially has long been the leading cause of death in Africa. Climate, economic factors, geographical location, human intervention and unstable security are factors influencing malaria transmission. Due to repeated infections and early interventions, the proportion of clinically atypical malaria or asymptomatic plasmodium carriers has increased significantly, which easily lead to misdiagnosis and missed diagnosis. African countries have made certain progress in malaria control and elimination, including rapid diagnosis of malaria, promotion of mosquito nets and insecticides, intermittent prophylactic treatment in high-risk groups, artemisinin based combination therapies, and the development of vaccines. Between 2000 and 2022, there has been a 40% decrease in malaria incidence and a 60% reduction in mortality rate in the WHO African Region. However, many challenges are emerging in the fight against malaria in Africa, such as climate change, poverty, substandard health services and coverage, increased outdoor transmission and the emergence of new vectors, and the growing threat of resistance to antimalarial drugs and insecticides. Joint prevention and treatment, identifying molecular determinants of resistance, new drug development, expanding seasonal malaria chemo-prevention intervention population, and promoting the vaccination of RTS, S/AS01 and R21/Matrix-M may help to solve the dilemma. China’s experience in eliminating malaria is conducive to Africa’s malaria prevention and control, and China-Africa cooperation needs to be constantly deepened and advanced. Our review aims to help the global public develop a comprehensive understanding of malaria in Africa, thereby contributing to malaria control and elimination.

## Introduction

Malaria is an infectious disease caused by *Plasmodium* species that affects humans and other animals. The main symptoms of malaria are fever, fatigue, vomiting, and headaches. In severe cases, malaria can cause jaundice, seizures, coma, or even death [1]. It is a prominent global public health issue. In 2022, there were 249 million cases of malaria worldwide resulting in an estimated 608,000 deaths [2]. Approximately 95% of the cases and deaths occurred in sub-Saharan Africa, and the most deaths are caused by *Plasmodium falciparum* (*P. falciparum)* [2]. Climate change, poverty, substandard health services and coverage, increased outdoor transmission and drug resistance are the reasons for the sustained outbreak of malaria in Africa, and they are also the huge challenges faced by Africans in combating malaria [3–5].

With the progress of drug development and the assistance of World Health Organisation (WHO), Africa has made certain progress in malaria control and elimination, including rapid diagnosis of malaria, promotion of mosquito nets and insecticides to block malaria transmission, intermittent preventive therapy (IPT), chemo-prevention in high-risk groups, and artemisinin based combination therapies (ACTs) for malaria treatment [6, 7]. Thus, since 2000, the mortality of malaria has fallen by 60% in the WHO African region [2]. However, about 300,000 African children still die from malaria every year, and much remains to be done to control the malaria epidemic in Africa.

In this paper, we summarize the current status of malaria in Africa by searching research papers through electronic databases of PubMed, MEDLINE, Web of Science and Embase up to March 30th, 2024. The search was focused on the epidemiological characteristics, risk factors, pathogenesis, diagnosis, prevention and treatment of malaria in Africa, as well as progress and challenges in the fight against malaria. To obtain the comprehensiveness of the search, we conducted an additional search for global malaria programmes reported over the past 20 years on the WHO website (https://www.who.int/). Through this review, we hope to help the global public fully understand malaria in Africa and propose measures to improve the status quo, with a view to contribute to the elimination of malaria in Africa.

## Epidemiological Characteristics of Malaria in Africa

### The Overall Prevalence of Malaria in Africa, as well as the Incidence and Mortality in Areas of High Endemicity

**Fig. 1:**
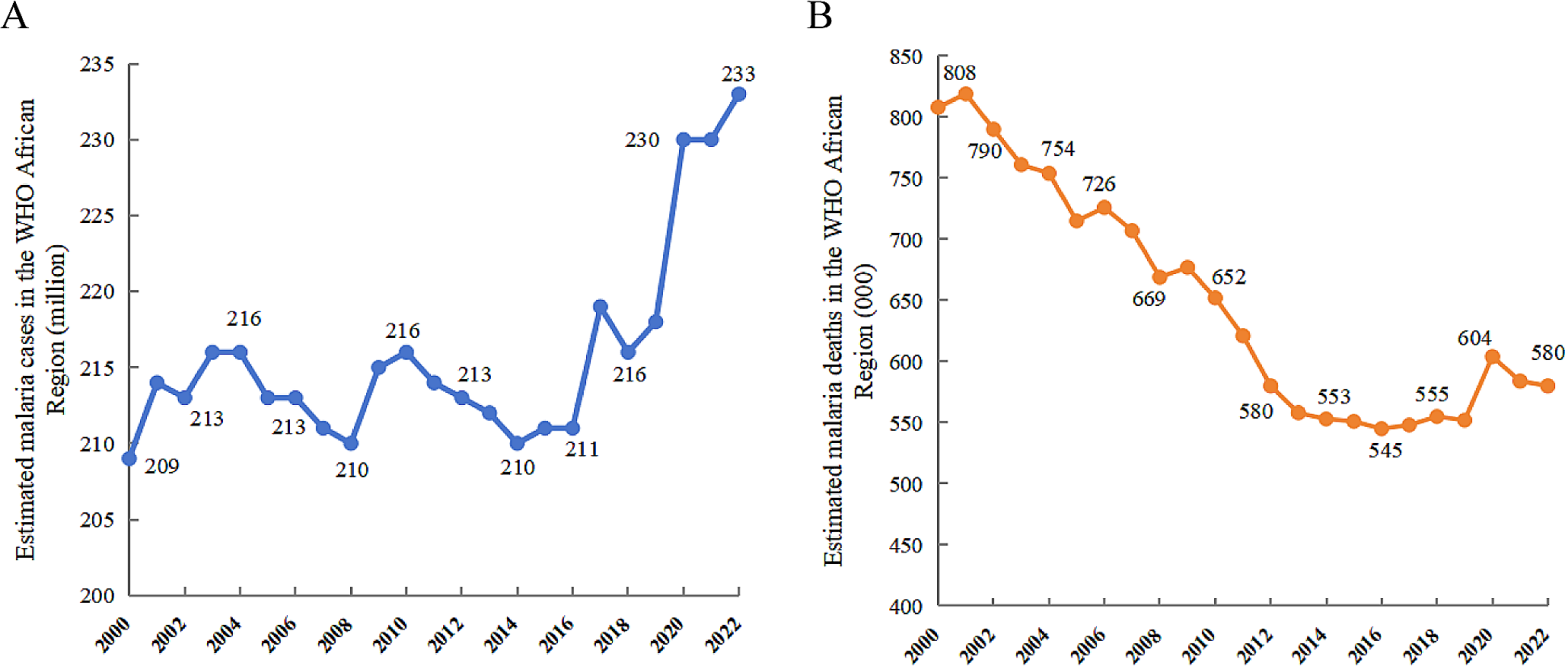
Estimated malaria cases and deaths in the WHO African Region from 2000–2022. (*Source: WHO estimates*)

Based on the World malaria report 2023, the data about malaria incidence and deaths in the WHO Africa Region are presented in Fig. 1. In 2022, there were an estimated 249 million cases of malaria worldwide, of which 94% (approximately 233 million cases) were from countries in the WHO African region [2]. Four countries– Nigeria (27%), the Democratic Republic of the Congo (12%), Uganda (5%) and Mozambique (4%)– accounted for almost half of all cases globally [2]. Malaria incidence in the WHO African Region reduced from 370 to 226 per 1,000 populations at risk between 2000 and 2019, but increased to 232 per 1,000 populations at risk in 2020, mainly because of disruptions to health services during the COVID-19 pandemic [8]. In 2022, malaria incidence in the WHO African region has declined to 223 per 1,000 population, due to the containment of the COVID-19 [2]. Africa also accounts for about 95% of global malaria deaths. Malaria deaths in the WHO African Region decreased from 143 to 57 deaths per 100,000 population at risk between 2000 and 2019, but increased to 61 in 2020. Similarly, estimated malaria related-deaths of WHO African Region decreased again to 56 per 100,000 population in 2022, which is consistent with the incidence [2, 8]. Four African countries occupy more than half of all malaria deaths globally, with Nigeria accounting for 31%, Democratic Republic of Congo for 12%, United Republic of Tanzania for 4% and Niger for 6% [2]. Malaria control has been largely achieved in some areas of Africa. In 2021, African countries, like Botswana, the Comoros, Eritrea, Eswatini, and Sao Tome and Principe all reported fewer than 10 deaths. And no malaria-related deaths have been reported in Harari Region and Cabo Verde since 2013 and 2015, respectively [9, 10].

### Species and Distribution of *Plasmodium*

There are five *Plasmodium* parasite species causing malaria in humans, including *P. falciparum*, *Plasmodium malariae* (*P. malariae*), *Plasmodium vivax* (*P. vivax*), *Plasmodium ovale* (*P. ovale*), and *Plasmodium knowlesi* (*P. knowlesi*) [11–14]. Among them, *P. falciparum* and *P. vivax* pose a huge threat to human health. As the main cause of malaria in Africa, *P. falciparum* has caused large number of deaths, accounting for more than 90% of the world’s malaria mortality. In 2020, 120.4 million pregnancies globally were at risk of infection with *P. falciparum*, and two-thirds of which (81.0 millions, 67.3%) occurred in areas of stable transmission [15]. *P. vivax* was previously considered to be dominant in most countries outside of sub-Saharan Africa, because people of African descent often lack the Duffy antigen receptor for chemokines. However, increasing evidences showed that *P. vivax* can transmit between Duffy-negative individuals [16, 17]. In some areas, *P. vivax* infection in Duffy-negative individuals is as high as 100% [18]. It has been demonstrated that a subset of Duffy-negative erythroblasts express Duffy antigen receptor for chemokines during terminal erythroid differentiation, which facilitates the invasion of *P. vivax* [19]. In addition, *P. vivax may* invade erythrocyte through other Duffy-independent pathways [20]. Due to its high temperature tolerance and ability to form dormant hypnozoites leading to relapse, *P. vivax* is enable to spread through the diverse African climate and outcompete *P. falciparum*, posing a serious health threat to Africa [21]. *P. malariae* is commonly seen in South America, Asia, and Africa. *P. malariae* only invades aged erythrocytes with a low incidence, and the severe disease rate is only 3% (95% confidence interval [CI]: 2-5%) [12]. *P. ovale* has been reported in Africa and Western Pacific countries, which affects 3 billion people and causes 1–3 million of deaths each year [13]. *P. knowlesi* has been detected in South East Asia, and males are the highest risk groups of getting infected, especially those working in forest [14]. But *P. knowlesi* is almost absent in Africa, probably because there are neither long-tailed macaques nor pig-tailed macaques, both of which are the host of *P. knowlesi*.

### Presusceptible Population

According to the World Malaria Report 2023 [2], malaria infection rates vary widely among populations. The susceptible population mainly includes the following categories: (1) infants and young children with imperfect immune system development [22]; (2) pregnant women without a strong immunity. Pregnant women are three times more exposed to malaria infection compared with non-pregnant women [23]; (3) pregnant women with partial immunity in the endemic area [24]; (4)pregnant women with partial incomplete immunity and HIV infection in endemic areas; (5) HIV-infected persons: the two often coexist together, leading to a worse prognosis; (6) an international traveler without immunity; (7) immigrants from non-endemic areas and their children.

Due to low acquired immunity, especially children younger than 5 years and pregnant women are the most susceptible populations [25]. It was estimated that the mortality rate of children under the age of five was as high as 70% in 2015 worldwide [26]. In 2021, in 38 moderate and high transmission countries in the WHO African Region, there was an estimated 40 million pregnancies, 13.3 million (32%) of which were exposed to malaria infection during pregnancy [27]. And malaria in sub-Saharan Africa kills 10,000 pregnant women every year, causing 8–14% of low-weight newborns and 3–8% of neonatal deaths [24]. Notably, since seasonal malaria chemo-prevention (SMC) is only conducted in children under 5 years of age in many areas, older siblings and parents who are not covered by SMC are more likely to become parasites reservoir [28, 29]. In Mali, malaria prevalence of children aged 5–15 years was higher than those younger than 5 years after SMC [30]. Attempts to extend the SMC intervention to children under 10 years of age have shown satisfactory results in multiple malaria-prone areas [31, 32].

### *Plasmodium* Infection Rate in Patients with Fever

The most typical symptom of malaria is fever. African countries have a high proportion of malaria among febrile people, especially children, in highly endemic areas [33, 34]. The correlation between fever and malaria infection in Africa is strong, but the association decreases with age, suggesting that anti-parasitic immunity may have some protective effect in adults [35]. Mabunda et al. [36] investigated 8,816 cases of children under 10 years old nationwide and measured the temperature simultaneously, and found 52.4% of patients with falciparum infection and 9.4% had fever. The proportion decreased with increasing age, peaking among children during the first 12 months of life (15.1%) [36]. Therefore, the current WHO guidelines for malaria control recommend empirical antimalarial treatment for children with fever in high endemic areas. Notably, malaria is routinely over-diagnosed and over-treated in Africa, especially in rural areas with limited medical resources, where many febrile patients are treated with anti-malarial drugs only based on clinical manifestations [37, 38]. A survey conducted in rural Uganda found that 62.0% of patients were misdiagnosed with malaria based on symptomatic diagnosis [38]. Rapid diagnostic test (RDT) for malaria and rational treatment are important to reduce malaria overdiagnosis and abuse of antimalarial drugs [39, 40].

### Asymptomatic Malaria Infection Rate in African Countries

There are a large number of asymptomatic infected residents in endemic areas of Africa, because they have developed partial immunity from repeated infections [41]. A large cross-sectional survey conducted in the western Kenyan highlands of Rachuonyo South reported that 93.2% (2,481/2,663) of malaria infections were asymptomatic [42]. The recessive infection of malaria accounts for a significant proportion in children [43]. Salgado et al. [43] investigated 1,354 healthy patients in three communities in western Kenya and found that children aged 11–15 years accounted for the greatest proportion total and sub-microscopic asymptomatic infections across all communities. Bousema et al. [44] reported a cohort study of 526 infants and children aged 6 months to 16 years in western Kenya, and found that the positive rate of gametocyte was 33.8% in children below 5 years of age, which gradually decreased with age. Without prompt and effective treatment, 18.6% of the untreated test-positive children developed gametocytaemia [44], suggesting that asymptomatic infected persons are at high risk of developing into symptomatic infections. Olliaro et al. [45] followed up post-treatment asymptomatic parasitemia for 28 days, found that 42% of total cases were still febrile, 30% had parasitemia but remained asymptomatic, only 23% had parasites cleared. Young age, parasitemia ≥ 500 parasites/µl, onset of parasitemia after day 14, and treatment with amodiaquine were risk factors for developing febrile symptoms [45]. Because asymptomatic infections are difficult to identify, they represent an important potential source and reservoir of malaria transmission in Africa and even the world.

## Factors Associated with Malaria Infection in High-endemic Areas in Africa

### Differences in Urban and Rural

Malaria rates vary greatly between urban and rural areas, due to huge differences in economic levels and health care conditions. Increased access to health care, education and prevention services in cities has led to lower rates of *Anopheles* bites, transmission intensity, and parasitic infection [46, 47]. It is reported that low socioeconomic status households were strongly associated with malaria hotspots (adjusted odds ratio [aOR] = 1.21, 95% CI: 1.03–1.40) in Burkina Faso [46]. Iqbal et al. [47] found that the infected population in rural (Dodowa) was significantly younger than that of urban (Accra) regions (median age 17 vs. 36 years, *p* < 0.0001). The incidence of parasitemia has significantly elevated in rural areas, especially among school-age children, as they usually spend more time outdoors and have more exposure to infected water and *Anopheles* mosquitoes [47]. However, another study took place in 1999 in Burkina Faso indicated that severe anemia (hemoglobin < 5 g/dl) was higher in rural patients (47.4% vs. 14.8%, *p* < 0.0001), while coma was higher in the urban patients (53.6% vs. 28.9%, *p* < 0.0001), which may be related to policy and economic conditions in different regions and periods [48]. In rural areas, seeking treatment outside often is delayed for the inability to pay for medical expenses [49].

### Nutritional Status

The infection and fatality rate of malaria are closely related to nutritional status. Jeremiah et al. [50] conducted a study about the relationship between malaria and nutrition in Port Harcourt, Nigeria, and found malnourished children under 5 years old were susceptible to malaria with high mortality rate (relative risk [RR] = 3.625, 95% CI: 1.81–5.43; *p* < 0.06). A cross-sectional assessment conducted in the North Region of Cameroon also supports a significant link between malaria and malnutrition in children (OR = 1.89, 95% CI: 1.12–3.19; *p* = 0.017) [51]. And anemia, low body weight and developmental delays are also common in them [51].

### Seasons and Climate

Climate and humidity are also important factors affecting the incidence of malaria. Tropical and subtropical areas are the most preferable by mosquitoes because the temperature range is between 15.4 °C and 35 °C [52]. Malaria-transmitting mosquitoes prefer areas with monthly precipitation of more than 80 mm and relative humidity of more than 60%. A large survey analysis of *Plasmodium* infection and socioeconomic factors in Burkina Faso indicated that rainfall and temperature were positively associated with malaria incidence [46, 53]. Another study conducted in Ouagadougou involving 3,354 children aged 6 to 12 years found that the overall blood anti-circumsporozoite (CSP) antibody positive rate and falciparum detection rate during the dry season were significantly lower than those during the rainy season (7.7% and 16.6% vs. 12.4% and 26.1%, respectively) [54]. Andrade et al. [55] finds that dry season limits the spread of *P. falciparum* parasites and low levels of parasites persist in the blood of asymptomatic Malian individuals during dry season, with minimally affecting the host immune response and rarely causing clinical symptoms. They believe that it is related to increased splenic clearance of longer-circulating infected erythrocytes [55].

### Geographical Location and Altitudes

In the highlands, different altitudes affect the survival of vectors in their breeding habitats, leading to divergent risks of malaria transmission and prevalence [56]. It has been shown that altitude is a major driver of malaria transmission in the western Kenyan highlands. The prevalence of malaria antibody was strongly related to altitude (1,350-1,600 m, *p* < 0.001) [42]. There also was a significant negative correlation between elevation and polymerase chain reaction (PCR) parasite prevalence [42]. Another cohort study involving 246 schoolchildren conducted in a highland area of western Kenya found that the prevalence and duration of *P. falciparum* infection decreased with age and altitude as well [57].

### Unstable Security

Malaria is a major health threat during and after war, due to the collapse of health systems, mass displacement of populations, and concentrated living in refugee camps prone to vector breeding [58, 59]. It has been shown that staying outdoors at night, standing water near houses and the number of children under 5 years old per household are risk factors for malaria in refugee [60]. Refugee children have higher in-hospital malaria mortality, partly due to delayed visits and higher rates of malnutrition [61]. Therefore, controlling unstable security is important to reduce the incidence of malaria. For populations in refugee areas, there is a need to promote the reduction of outdoor stays at night, the elimination of standing water and the use of insecticide-treated mosquito nets, especially considering the number of children under 5 years of age per household [60]. For patients with severe malaria, rapid diagnosis and referral are needed to reduce malaria mortality [61].

## The Pathogenic Mechanism of Malaria

Malarial infection begins when a person is bitten by an infected female *anopheles* mosquito and *Plasmodium* spp. in the form of sporozoites are injected into the bloodstream. The sporozoites travel to the liver and reproduce asexually over the next 7–10 days. There are no symptoms during this period. The parasites, in the form of merozoites, emerge from the liver cells in vesicles and travel through the heart to the capillaries of the lungs. The vesicles eventually disintegrate and release the merozoites to the bloodstream where they invade and multiply in erythrocytes, causing destruction of red blood cells and anemia [62]. *P. vivax* mainly invades senescent red blood cells, so anemia is less obvious [63]. *P. falciparum* influences red blood cells of different ages with high infection density, so anemia appears earlier and more prominent [64].

After the malaria parasite and its decomposition products enter the human body, macrophages, immune cells and inflammatory factors are rapidly activated and participate in the process of removing pathogens and red blood cell debris, so patients often have splenomegaly and hypersplenism [65]. During initial infection, the ingestion of merozoites and fragments by macrophages or the presence of antigen-presenting trophozoites in the circulation or spleen leads to the release of tumor necrosis factor-alpha (TNF-α) [66]. Then, TNF-α induces aggregation of interleukin-10 and γ-interferon, which mediates an inflammatory cascade, causing fever during infection [67, 68]. Subsequently, the acquired immune system is activated, and a certain degree of antibody cascade produced by the macrophage-T cell-B cell axis amplifies macrophage activity, resulting in more efficient removal of the parasite [69, 70]. With sustained immune stimulation, additional antibodies bring more protection, which explains some asymptomatic infections.

Cerebral malaria (CM) is a serious type of *Plasmodium* infection and occurs more frequently in *P. falciparum*, which is mainly related to the red blood cells in the brain capillaries [71]. *P. falciparum* attaches to capillary endothelial cells through *Plasmodium falciparum* erythrocyte membrane protein 1 (PfEMP1) and causes local capillary obstruction and cell hypoxia through cross-agglutination and adsorption, which can cause severe brain edema and brain cell damage, leading to severe headache, delirium, and even coma [72]. In addition, parasite-infected erythrocyte specific protein 2 (PIESP2) is also involved in mediating CM by sequestrating infected red blood cells, inducing the inflammation response, and impairing the integrity of blood-brain barrier. PIESP2 is a *P. falciparum*-specific immunogenic protein, which expression begins at the early trophoblast stage and gradually increases with the development of the parasite [73].

When macromolecular substances such as hemoglobin and antigen/antibody complexes block the glomerular basement membrane, it can cause acute immunoallergy. And patients often have soy sauce-like meurine, oliguria/anuria, and acute renal failure manifestations with a sharp increase in creatinine and urea nitrogen, known as hemolytic uremic syndrome [74]. It is one of the most dreaded complications of severe malaria.

In addition, VAR2CSA, the variant of PfEMP-1, binds to chondroitin sulfate-A in the placenta and evades host immune recognition, preventing immune activation of the parasite and spleen clearance, thus pregnant women are more susceptible to malaria than non-pregnant women [75, 76]. In addition, parasitemia in placental malaria induces immune imbalances, as well as inflammation, which disrupt placental function and deplete fetal nutrients [77, 78]. Therefore, low-weight infants and stillbirths among pregnant women infected with malaria are very common.

## Diagnosis of Malaria

The diagnosis of malaria depends on epidemiological history, clinical symptoms, and laboratory tests [79].

### Clinical Manifestations

Due to differential pathogenic characteristics, *Plasmodium* parasites have certain differences in incubation period and clinical manifestations [79], as shown in Table 1. According to the clinical symptoms and severity of comorbidities, the patients can be divided into normal and severe malaria. Ordinary infected persons may have fever, chills, sweating, headache, nausea, muscle pain, and vomiting. Typical symptoms of the onset last 6–10 h, with no symptoms during intermittent period. But, there was no obvious pattern of onset in atypical patients. The younger the children, the less typical the symptoms, can appear stunting, malnutrition, anemia, giant spleen and other manifestations [80]. Pregnant women with atypical symptoms may have miscarriage and stillbirth. In severe cases, malaria may cause jaundice, seizures, coma, kidney failure, and even death, known as severe malaria [1]. All five *Plasmodium* species can cause severe malaria, but most deaths are caused by *P. falciparum*, the cerebral form of which is most common. Specific diagnostic criteria for severe malaria are shown in Table 2.

**Table 1:** Characteristics of different types of malaria infection

Plasmodium species	Incubation period, days	Interval between outbreaks		Hypnozoite stage	Relapse	Recrudescence	Main epidemic areas
*P. falciparum*	9–14		within 24 h without regularity	No	No	Yes	Sub-Saharan Africa, South and Southeast Asia, Eastern Mediterranean, Western Pacific, South America
*P. vivax*	11–14		every 48 h	Yes	Yes (6–12 months) (> 2 years in some cases)	Yes	Similar to *P. falciparum* and also present in the Korean Peninsula
*P. ovale*	16–18		every 48 h	Yes	Yes (8–45 months)	Yes	Sub-Saharan Africa, Southeast Asia, Western Pacific
*P. malariae*	18–40		every 72 h	No	No	Yes	South America, Asia, Africa
*P. knowlesi*	9–12		within 24 h without regularity	No	No	Yes	Southeast Asia

**Table 2:** Diagnostic criteria for severe malaria

Plasmodium species	Criteria	
	Signs and symptoms	Laboratory and radiology
*P. falciparum*	1.Impaired consciousness: Glasgow Coma Scale score < 11, or Blantyre Coma Scale < 3 in children	1.Acidosis: base deficit of > 8 mEq/L or plasma bicarbonate < 15 mEq/L or venous plasma lactate ≥ 5 mmol/L
	2.Convulsions (> 2 within 24 h)	2.Anemia: hemoglobin concentration < 7 g/dL or hematocrit < 20% with a parasite count > 10 000/µL
	3.Respiratory distress (acidotic breathing)	3.Hypoglycemia (blood glucose < 2.2 mmol/L or < 40 mg/dL)
	4.Prostration: unable to sit, stand, or walk without assistance	4.Parasitemia: ≥5%
	5.Significant bleeding: recurrent or prolonged bleeding from the nose, gums, or venipuncture sites; hematemesis or melena	5.Jaundice: plasma or serum bilirubin > 3 mg/dL and parasite count > 100,000/µL
	6.Jaundice	6.Renal impairment (creatinine > 265 mmol/L [3 mg/dL] or blood urea > 20 mmol/L)
	7.Anuria	7.Pulmonary edema: radiologically confirmed or oxygen saturation < 92% on room air with a respiratory rate > 30/min
	8.Hemoglobinuria (blackwater)	
	9.Repeated vomiting	
*P. vivax*	Defined as for falciparum malaria but with no parasite density thresholds	
*P. knowlesi*	Defined as for falciparum malaria	except as below: *P. knowlesi* parasite density > 100,000/µL; Jaundice and parasite density > 20,000/µL

Notably, some patients with *P. vivax* or *P. ovale* may relapse, which is related to the presence of dormant or delayed sporozoites of malaria parasites in the liver [81]. Therefore, the treatment of *P. vivax* and *P. ovale* should be supplemented with drugs against intrahepatic *Plasmodium* in addition to drugs against intraerythrocytic *Plasmodium*. *P. falciparum*, *P. malariae*, *P. knowlesi* usually do not recrudesce owing to the absence of dormant seeds in their extracellular phase. All kinds of *Plasmodium* parasites can reignite, which is related to incomplete drug treatment in the intraerythrocytic phase, often appearing within 1 month after clinical cure.

### Etiological Examination

#### Microscopic Detection of Peripheral Blood Smear

*Plasmodium* parasites can be identified by examining a drop of a patient’s blood under a microscope with Gilles or Reye stain [82]. This method is intuitive and clear, can identify the species and density of *Plasmodium* parasites, contribute to the early detection of severe malaria, and is the “gold standard” recommended by WHO for malaria diagnosis [83]. But the accuracy of the test depends on the proficiency of the microscopist. Automated microscopy and digital malaria microscopyare have been developed to improve diagnostic performance [84, 85].

#### RDT

RDT is characterized by simple and rapid detection. The results showed that the sensitivity and specificity of RDT in the diagnosis of *P. falciparum* were 92% and 96.6%, and those of *P. vivax* were 72.9% and 99.1%, respectively, compared with microscopy [83]. RDT is a reliable alternative for the diagnosis of malaria, especially in remote areas where qualified microscopists are scarce [86]. However, the performance of RDT is affected by batch, brand, ability to cross the membrane, specificity and sensitivity of the antigen-antibody complex [87]. For example, the diagnostic strip with histidine rich protein (HRP)2/HRP3 as the target antigen showed high sensitivity and specificity for *P. falciparum*, but could not detect other *Plasmodium* strains [88]. The diagnostic test strips with lactate dehydrogenase (LDH) as the target antigen can detect *P. falciparum* or non-*P. falciparum*, but cannot distinguish the species of non-*P. falciparum*, and was less sensitive to low protozoa density [87]. In addition, gene deletions of pfHRP2 and pfHRP3 in *P. falciparum* are increasingly prevalent, even up to 86.5% in some areas [88]. *P. falciparum* infections by parasites with both pfHRP2 and pfHRP3 deletions were tested HRP2-negative by RDT, which lead to a large number of missed diagnoses [88, 89]. Moreover, RDT cannot monitor response to antimalarial therapy. New highly sensitive RDTs are developing to overcome these deficiencies [90, 91]. Wherever possible, PCR and microscopy should be used to confirm and monitor malaria.

#### *Plasmodium* Gene Detection

Using PCR to copy the deoxyribonucleic acid (DNA) can not only identify the species of *Plasmodium*, but also detect the genes related to drug resistance [92, 93]. This method facilitates the detection of misdiagnosed submicroscopic and asymptomatic individuals using RDT and microscopy, with specificity and sensitivity up to 88-94% and 98-100%, respectively [94]. However, it is very expensive, requires specialized equipment and experienced experts, and is not suitable for large-scale deployment in Africa.

#### Biosensors

Biosensors rely on biochemical interactions between biological components and the transducer matrix and analytes (PfHRP-2, pLDH, aldolase, and biocrystalline heme) [95, 96]. The transduction characteristics of the sensor are changed by this reaction, as a positive or negative correlation of temperature, absorbance, or conductivity, and thus the analyte concentration is deduced [95, 97]. Compared to the common RDT, the sensitivity and specificity of the automated fluorescent blue-ray device-based malaria diagnostic equipment were 98.1% and 54.8%, respectively [98]. The device can also detect asymptomatic people [95, 97]. Biosensor is still less used, but it seems to be a promising detection technique.

## Preventive Measures against Malaria in African Countries

As an infectious disease, malaria transmission can be blocked by good management, cutting off the transmission route, and protecting the susceptible population. But how to block these three links is influenced by many factors, such as natural environment, humanities, economic environment and so on. Comprehensive preventive measures are needed to block malaria. In malaria endemic countries, certain protection against malaria should be offered to vulnerable groups including pregnant women, under-five children, nomadic populations, and visitors.

### Strengthening Publicity and Improving the Awareness of Malaria Prevention

To prevent malaria, awareness is the key. The quality, living environment and religious beliefs of the residents in African countries are different. People in some areas think that only those who are sick need to take medicine, while ignoring the risk of occult infection. The government propaganda and policy promotion is crucial for the prevention of malaria. Active case detection is recommended by WHO. And there is a need to strengthen social interaction. It is showed that mothers and pregnant women can learn about the benefits of malaria prevention behaviors through conversation or direct observation from neighbor’s experiences with mosquito nets or preventive treatment during pregnancy, thus reducing the risk of infection in pregnant women and children [99].

### Timely Monitoring and Treatment of Malaria

In recent years, in many parts of Africa, malaria epidemiological surveillance platforms and parasite genetic testing platforms have been established based on the national health system in schools and other places [100–102]. With targeted monitoring measures and treatment of malaria, the prevalence of malaria has been significantly reduced. However, the contribution of asymptomatic patients to malaria transmission needs to be vigilant, as this group is huge and easily overlooked.

### Comprehensive Environmental Management and Chemical Drug Spraying

Many areas of South Africa have tropical rainforest climates, with a dry season and a rainy season. The rainy season is hot and humid, and the environment is surrounded by strong trees and thick grass, which create favorable conditions for mosquito breeding and growth. Therefore, comprehensive management of the residential area by eradicating the surrounding weeds and shrubs, leveling the camp site, dredging ditches, clearing the water in the depression and the use of larval agents is beneficial for reducing larval breeding [103, 104]. And adult mosquitoes could be controlled through indoor residual spraying (IRS) and insecticide-treated nets (ITNs) [105, 106]. Compared to equivalent populations with no nets, ITNs reduced child mortality by 17%, parasite prevalence by 13%, uncomplicated malaria episodes by 50%, and severe malaria by 45% [105]. A recent meta-analysis reported that IRS reduced the risk of malaria by 65% [107]. However, the increasing resistance of insecticides reduces the effectiveness of IRS and ITNs [108], pose a growing threat to malaria control. Chemoprophylaxis is also the primary route of prevention, mainly for people who have traveled to malaria-endemic countries [109]. The choice of drugs depend on the travelling destination, the duration of potential exposure to vectors, parasite resistance patterns, season, and susceptible population. In endemic countries, SMC has been recommended for autochthonous infants, young children and pregnant women, depending on endemicity level and seasonality of transmission [110, 111]. A cluster-randomised trial conducted in twenty-four villages of south-east Senegal demonstrated that SMC for children under 10 years of age given over 5 months was feasible, well tolerated, and effective in preventing malaria episodes. Besides, SMC reduced the prevalence of parasitemia and anaemia in children [112]. The most common drugs used for chemoprophylaxis include Sulfadoxine-pyrimethamine plus amodiaquine, atovaquone-proguanil, doxycycline, and mefloquine [112, 113].

### Regulating Individual Behavior and Selecting Appropriate Protective Measures

Personal protection can be divided into drugs and physical protection. Drug protection is divided into oral preventive drugs and in vitro repellent drugs. People traveling in areas with malaria cases are advised to use antimalarial drugs: mefloquine, chloroquine, malarone (atovaquone-proguanil), primaquine. The combination of physical and chemical approaches has been proven to be more effective against malaria, reducing the adverse drug reactions and the occurrence of drug resistance to *Plasmodium* [112]. Personal protective measures to reduce the risk of getting bites by mosquito include the mosquito bed nets (preferably ITNs) [105], sleeping or resting in screened or air-conditioned rooms, wearing clothes to cover most of the body when going outside [114], and using mosquito repellent [115].

### Vaccine Development and Piloting

To overcome the threat of drug resistance, multiple vaccines are being actively developed and clinically studied [116]. RTS, S/AS01 vaccine is the most extensively tested vaccine and as a candidate for prevention of *P. falciparum* by inducing the production of anti-CSP IgG antibodies [117]. Phase 3 clinical trials conducted in 11 African sites shows that the RTS, S/AS01 vaccine has a protective efficacy of 46% in children during the 18 months after vaccine dose 3 (per protocol) [116]. But, the protection of vaccine is partial, wanes over time, and may be also depending on age. In both infants aged 6 to 12 weeks and young children 5 to 15 months old, vaccine efficacy waned rapidly [116]. And the protection was lower in infants of 6–12 weeks than in young children of 5–17 months old. For kids who received the first three vaccinations in a period of 0-1-2 month schedule, the incidence of clinical malaria has been reduced by 51% over the first year of follow-up. And the efficacy dropped to 26% over 48 months of follow-up [118]. Clinical trials in Mali and Burkina Faso show that providing children the vaccine just prior to high transmission seasons results in substantial reduction in the incidence of clinical malaria and severe malaria, and achieves better effectiveness in combination with SMC [119]. At present, RTS, S/AS01 is recommended by the WHO for active immunization against malaria in a schedule of 4 doses to children from 5 months of age [120]. For countries with areas of highly seasonal transmission of malaria, an optional alternative 5-dose seasonal delivery strategy can be used to optimize vaccine efficacy [120].

R21/Matrix-M is the second malaria vaccine officially recommended for children in high-transmission settings by the WHO [121]. Much like RTS, S/AS01, R21/Matrix-M is also based on the fusion of C-terminal portion of the CSP from *P. falciparum* to the N-terminus of HBsAg [117]. Nevertheless, the R21 is combined with the Matrix-M proprietary adjuvant and does not include unfused HBsAg, allowing for a higher concentration of CSP at a lower dose [122]. R21/Matrix-M vaccine is well tolerated, with low frequent adverse events and no treatment-related deaths. The 12-month vaccine efficacy was 75% (95% CI: 71–79%; *p* < 0.0001) at the seasonal sites and 68% (95% CI: 61–74%; *p* < 0.0001) at the standard sites before first clinical malaria episode [123]. Similarly, vaccine efficacy against multiple clinical malaria episodes was 75% (95% CI: 71–78%; *p* < 0.0001) at the seasonal sites and 67% (95% CI: 59–73%; *p* < 0.0001) at standard sites [123]. The efficacy of R21/Matrix-M vaccine also wanes moderately [123]. But overall, R21/Matrix-M is efficient, low-cost and has been licensed in many African countries [121, 124].

According to the prediction, at 100% vaccine coverage, 5.2 million cases (95% CI: 3.5–8.2 million) and 27,000 deaths (95% CI: 14,000–43,000) in children younger than 5 years could be averted annually at a dose constraint of 30 million, and 10.0 million cases (95% CI: 6.7–15.7 million) and 51,000 deaths (95% CI: 25,000–82,000) at a dose constraint of 60 million [125]. However, there are difficulties such as low acceptance, lack of funds to promote the vaccine in Africa, and how to maintain longer protective efficacy [126]. Novel vaccine delivery approaches are nowadays being advanced, including self-amplifying RNA vaccine delivery, self-assembling protein nanoparticle methods, CSP protein-based approaches, and whole organism vaccination [127]. Other novel vaccines, including GMZ2, the *Plasmodium falciparum* sporozoite (PfSPZ) vaccine and so on, are also currently in clinical development [128, 129].

## Drug Treatment for Malaria

Malaria treatment includes etiology treatment, symptomatic and complication treatment, and necessary supportive therapy to maintain homeostasis and protect core organ function [79, 130]. Among them, etiological treatment is crucial, which requires the selection of antimalarial drugs with fewer adverse reactions to kill the parasite quickly and avoid long-term recurrence. Drugs used to kill intraerythrocytes (control clinical symptoms) includes Chloroquine phosphate, Piperaquine phosphate, Linalidine phosphate, Artemisinin drugs. Drugs killing *Plasmodium* in intrahepatic stage (control recurrence and stop transmission) is primaquine phosphate. Details were shown in Table 3.

**Table 3:** Antimalarial drugs

Medicine	Function characteristics	Function stage	Note
Sulfadoxine/pyrimethamine	an antifolate antimalarial drug; Sulfadoxine targets *Plasmodium* dihydropteroate synthase; Pyrimethamine inhibits dihydro-folate reductase.	intrahepatic stage, gametocyte stage	It has a high rate of drug resistance and can be used in combination with other anti-malarial drugs.
Chloroquine phosphate	4-aminoquinolines drug; enriched in diseased red blood cells; rapid and well absorbed orally; its concentration in red blood cells is 10–20 times higher than in plasma.	asexual blood stages of various *Plasmodium* parasites	not recommended for falciparum treatmen
Piperaquine phosphate	4-aminoquinolines drug; well absorbed orally; stored in the liver, and then gradually released into the blood, with a plasma half-life of up to 28 d	asexual blood stages of various *Plasmodium* parasites	cross-resistant to chloroquine phosphate
Linalidine phosphate	a new antimalarial drug of benznaphidine	asexual blood stages of various *Plasmodium* parasites	It can be used in the treatment of chloroquine phosphate -resistant falciparum malaria.
Artemisinin drugs	a new sesquiterpenoid lactone antimalarial drug extracted from the traditional Chinese medicine Artemisia annual	asexual blood stages of various *Plasmodium* parasites	(1) Artesunate injection: a water-soluble derivative of artemisinin, recommended as the treatment of choice for severe malaria; (2) Artemether injection: a lipid-soluble derivative of artemisinin, recommended as one of the alternative treatments for severe malaria in areas without artesunate injection; (3) ACTs: include artemether/alcohol, artesunate/amodiaquine, dihydroartemisinin/piperaquine phosphate, artesunate/Norine, artesunate-mefloquine and artesunate-sulfamidoxine-pyrimethamine, which shorten the course of artemisinin treatment and delay the development of resistance.
Primaquine phosphate	rapidly and completely absorbed orally, with a plasma half-life of only 5 to 6 h	intrahepatic stage, gametocyte stage	It is often used in combination with erythroid *Plasmodium* drugs for the radical treatment of malaria with *P. vivax* and *P. ovale* infections. In the falciparum endemic zone, a single dose of low-dose primaquine phosphate has also been recommended in combination with ACTs for falciparum therapy and is not affected by G6PD deficiency.

ACT: artemisinin based combination therapy

### Treatment of Non-severe Malaria

Except for early pregnancy, ACTs are recommended as the first line treatment for uncomplicated *P. falciparum* infection in all populations. Quinine plus clindamycin 7-day treatment is the recommended method for uncomplicated *P. falciparum* infection in early pregnancy [2]. ACT is also effective against non-*P. falciparum*, therefore it is recommended for the treatment of mixed and non-specific infections [131]. In the event of standard ACT treatment failure, it can be administered together with artesunate and primaquine. The uncomplicated *P. vivax*, *P. ovale*, and *P. malariae* obtained in chloroquine sensitive areas can be treated with chloroquine. Adults and children with uncomplicated *P. vivax*, *P. ovale*, *P. malariae*, and *P. knowlesi* infections should be treated with ACT containing piperaquine, mefloquine, or phenylfluorenol (excluding early pregnancy) [79].

### Therapy of Severe Malaria

Severe malaria has a high mortality rate, which requires comprehensive rescue measures of both etiology and symptomatic treatment, and timely transfer to intensive care unit for treatment when necessary. Artesunate is the first drug and has been shown to significantly reduce mortality of severe malaria. Compared with quinine (previous first-line treatment), the mortality of children in Africa was decreased by 22.5% (95% CI: 8.1–36.9%) after Artesunate treatment [132]. If there is no artesunate injection, artemether injection can be used intramuscularly. It is important to note that artemether is absorbed more slowly and is more unstable than water-soluble artesunate, especially in patients with shock. Intravenous infusion of pyronaridine phosphate also can be used for treatment. The dosage and usage of above drugs are shown in Table 4. For the complications of severe malaria, such as high fever, brain edema, acidosis, respiratory failure, anemia, liver and kidney function injury et al., antipyretic drugs, mannitol dehydration, sedation, sodium bicarbonate acid correction, diuresis, blood transfusion, respiratory function support, and nutritional support are required. If necessary, blood purification, ventilator-assisted support therapy, and antibiotics should also be given in time [79].

**Table 4:** Etiological treatment of severe malaria

Drug	Recommendation	Route of administration	Specific usage	Note
Artesunate	preferred treatment	intravenous injection	adults: 0, 12 and 24 h each, 120 mg/time (2.4 mg/kg); children with body mass < 20 kg: 3 mg/kg, once a day, 120 mg/time for at least 7 days.	When the patient regains consciousness and restores to eat, the artesunate injection can be stopped and replaced with compound artemisinin for 1 course of treatment. The compound artemisinin can be extended if necessary until the parasites disappear.
Artemether	alternative treatment	intramuscular injection	adults: the first dose is 160 mg, followed by 1 dose/day, 80 mg/time, or first dose 3.2 mg/kg, later 1.6 mg/kg for at least 7 consecutive days; children: the usual injection dose is similar to that for adults (calculated according to kg of body weight).	If the patient is awake and able to eat, at least 1 course of consolidation therapy with compound artemisinin can be taken until the parasites disappear.
	alternative treatment	intravenous infusion	adults: once a day, 160 mg/time, for 3 consecutive days; children: the usual injection dose is similar to that for adults (calculated according to kg of body weight).	Intravenous injection is strictly prohibited. It should be used with caution in patients with serious heart, liver and kidney diseases.

### Potential Novel Antimalarial Drug Candidates

However, emerging drug resistance has prompted an urgent search for new antimalarial drugs. It has been shown that many marine natural products, such as paenidigyamycin A, tedania braziliensis, ceratinadin E, have strong antimalarial effects, but their clinical application is limited by funding, experimental conditions and the amount of compounds [133]. Selective *P. falciparum* histone deacetylase inhibitor is also an emerging potential antimalarial agent, but it is still in the preclinical research stage due to its toxicity [134]. Moreover, OZ439, DSM265 or OZ439/DSM265 combination therapy can be promising alternatives for treating uncomplicated *P. falciparum* and *P. vivax* infections [135–137]. OZ439, also known as artefenomel, is a novel antimalarial drug with a mechanism similar to artesunate but with a longer half-life [137, 138]. DSM265, another novel synthetic antimalarial drug, kills the parasite by inhibiting *Plasmodium* dihydroorotate dehydrogenase, which is an important enzyme for pyrimidine biosynthesis in parasites [135]. DSM265 is a single-dose regimen with a long elimination half-life (up to 100 h) and satisfactory tolerance [139]. Both OZ439 and DSM265 have entered the clinical phase 2 studies [138, 140].

## The Progress Fight against Malaria in African Countries

Since the launch of the Repel Malaria Initiative and the United Nations Millennium Development Goals (MDGs) in 2000, international funding for malaria control in the sub-Saharan continent has increased nearly 20 times [141, 142]. Subsequently, the African Leaders Malaria Alliance (ALMA) was established and declared 25th of April as “Africa Malaria Day”. Since that period, many advances of malaria control have been achieved, including ACTs for malaria treatment, rapid diagnostic of malaria, long-lasting insecticide treated nets (LLINs), and the development of vaccines. Although the MDGs, set in 2000 to achieve a 75% reduction in malaria incidence in Africa, have not yet been met, the results have been remarkable. From 2000 to 2022, the rate of falciparum malaria mortality in the sub-Saharan desert continent has been halved, and the incidence of malaria with significant clinical symptoms has decreased by 40% [2]. It means that about 663,000,000 clinical cases have been prevented. It is estimated that a 10% decrease in incidence is attributed to the use of IRS, 22% to ACT programs, and 68% was associated with the use of ITNs [143].

Cooperation with other countries has also contributed significantly to malaria control and elimination in Africa and made certain achievements. For example, China, which has achieved zero malaria, has carried out long-term Sino-African medical and health cooperation in the field of malaria with African countries, including sending medical teams, carrying out training programs and joint drug research, donating drugs and medical equipment, assisting in disease network surveillance, and conducting academic exchanges [141, 144, 145]. With the 85 rounds of 1,7-malaria Reactive Community-based Testing and Response (1,7-mRCTR), the conduction of China-UK-Tanzania tripartite pilot project reduced the odds of malaria infection in the intervention wards in southern Tanzania by 66%, which significantly eased the local malaria burden [146]. These indicate that China’s systematic model and strategy of malaria surveillance and response are worthy of reference for endemic countries in Africa.

## Ongoing Malaria Challenges in African Countries

Although remarkable progress has been achieved in the fight against malaria, the outbreak of COVID-19 has led to a renewed increase in malaria, which remains a major burden of disease in many African countries [8]. It should not be ignored that about 300,000 African children die of malaria each year. Following challenges need to be actively addressed to control the malaria pandemic in Africa.

### Climate Change and Destruction of the Natural Environment

The tropical and subtropical climate of sub-Saharan Africa is suitable for the ancient malaria vectors, *Anopheles gambiae*. Therefore, malaria is prone to outbreak in Africa. Malaria is low transmissible in plateau areas due to low temperatures [147]. However, in recent years, due to frequent changes in the climate, Africa has experienced continuous temperature rise, accelerated sea level rise and extreme weather, resulting in more and more malaria in the plateau region [148]. Fortunately, many areas are being monitored as quickly as possible to control the outbreak of malaria. Studies have confirmed that deforestation and other actions destroying the natural environment also promote malaria. Deforestation expands areas of sunny and stagnant water, increasing mosquito larval habitat [149]. Anyway, excessive deforestation is undesirable and not conducive to ecological balance.

### Poverty, Substandard Health Services and Coverage

On the African continent, there is a vicious cycle of poverty and malaria [150]. Housing conditions and poor living environments are factors of malaria exposure [151]. Due to the low level of economic development of most African countries, health services and coverage are substandard, and the population has limited access to diagnostic services and effective treatment. Government funding for vector control, disease diagnosis and surveillance is inadequate, and some areas even rely on donations to fight malaria, resulting in persistently high morbidity and mortality [152]. Poverty is one of the main challenges facing malaria in Africa, as well as the survival of Africans.

### Controlling Cross-border Malaria

Malaria is a regional problem, but not a country-specific one. Populations are constantly on the move, and areas with low transmission or eradication need to be mindful of the challenge of imported malaria from areas with high transmission. Many neighboring countries have achieved mutual benefit and win-win results through cooperation, like Lobamba Spatial Development Initiative between Mozambique, South Africa and Swaziland, which brings an 80% reduction in malaria prevalence in Mozambique and a 98% reduction in South Africa and Swaziland [153]. Similarly, the implementation of the MoSaSwa (Mozambique, SA and Swaziland) initiative, the Trans-Kunene Malaria initiative and the Zim-Zam initiative, have also significantly reduced the burden of malaria in the participating countries [154, 155].

### Increased Outdoor Transmission and the Emergence of New Vectors

In response to long-term insecticide pressure, vectors have changed their biological behavior and bite hosts outdoors more and earlier to avoid exposure to pesticides [156]. There is an urgent need to develop effective means of prevention against outdoor transmission. The Centres for Disease Control light trap and the human decoy trap may be a good method for outdoor insect trapping and surveys [157]. The emergence of new vectors contributes to low levels of outdoor transmission, since traditional insecticidal methods only target *Anopheles gambiae* (*s.s*), *An. arabiensis* and *An. funestus*. Researches on new vectors also are necessary to carry out.

### Resistance to Antimalarial Drugs and Insecticides

One of the biggest threats to eliminating and eradicating malaria is the emergence of insecticide and drug resistances. Both the malaria vectors and parasites have proved to be very adaptable. They have rapidly developed mechanisms to survive and multiply in the presence of insecticides and antimalarial drugs, including biological behavior changes and genetic mutations [158]. Insecticide resistance is widespread across the African continent. It reduces the efficacy of strategies based on suppressing vectors, such as LLINs and IRS [159]. The next-generation ITNs are trying to popularize universally to change the status quo. Pirimiphos-methyl is a better alternative than pyrethroids for IRS in areas with widespread of pyrethroid resistance [160]. In addition, local insecticide resistance monitoring may be necessary to select highly sensitive insecticides prior to implementation of IRS [108]. There are also studies attempting to combine ITN and IRS to maximize the benefits of both approaches [159, 161].

Resistance to antimalarial drugs is also very troublesome and has been reported in almost all malarial regions [162]. At present, the failure rate of chloroquine and sulfadoxine-pyrimethamine in malaria treatment is very high in many areas of Africa, and even Artemisinin resistance has also emerged [158, 163, 164]. The emergence of drug resistance is closely related to the abuse of antimalarial drugs and the imperfect course of drug treatment [165]. In addition, genetic mutation of *Plasmodium* is also the main mechanism of drug resistance. In the context of SMC, there are several mutations in genes of *P. falciparum* affecting the metabolism of sulfamidoxine-pyrimethamine and amodiquine, including Pfcrt (*Plasmodium falciparum* chloroquine-resistant transporter), Pfmdr1 (*Plasmodium falciparum* multidrug resistance 1), DHFR (dihydrofolate reductase) and DHPS (dihydrosphenoate synthetase) genes [166, 167]. And Kelch 13 (K13)-propeller mutations were identified as a major determinant of artemisinin resistance [168]. Drug resistance leads to poor efficacy of chemo-prevention and first-line drug therapy. Studies have shown that rotating or alternating the use of antimalarial drugs could reduce resistance. Alternatively, drugs with different resistance characteristics can be deployed in parallel. Boni et al. [169] proposes a strategy of “multiple first-line therapies” to maintain the efficacy of therapeutic drugs. Triple ACTs, combining an artemisinin and two existing partner drugs, could be one of the last remaining safe and effective treatments for multidrug-resistant *P. falciparum*. In addition, matching and combining long-acting and short-acting partner drugs according to their pharmacokinetic characteristics is also a potential mean to reduce drug resistance [170, 171]. Governments, researchers and clinicians should try and scale up these novel strategies to address drug-resistant problem.

## Conclusion

At present, malaria remains a major disease burden on the African continent, especially falciparum malaria, which is the highly lethal. With the help of the international health organizations, Africa has launched a series of effective anti-malaria measures, which have greatly reduced mortality and morbidity of malaria in Africa. However, due to Africa’s special climate and poor living conditions, population mobility, and increasing drug and insecticide resistances, fighting against malaria faces many difficulties and challenges. To achieve MDGs of the United Nations, African governments and even international organizations need to mobilize more funds to strengthen basic health facilities and services in Africa, improve malaria detection, strengthen surveillance and treatment coverage, and advance drug and vaccine development. There is a need for African governments to provide IRS and LLINs to all people at risk, especially for African women and children. Free testing and treatment should be fully available for pregnant women and children infected. For external travelers and aid providers, free testing and oral prophylactic drugs also should be available to help control the global spread of malaria. China’s experience in eliminating malaria is conducive to the fight against malaria of Africa, and it is necessary to continue and deepen China-Africa cooperation. Our article systematically reviews the epidemiology, diagnosis, prevention and treatment, progress and challenges of malaria in African countries, with a view to helping the global public develop a comprehensive understanding of malaria in Africa and promoting the elimination of malaria in Africa and even the world.

## Data Availability

No datasets were generated or analysed during the current study.

## Abbreviations

*P. falciparum*: *Plasmodium falciparum*
WHO: World Health Organisation
IPT: Intermittent preventive therapy
ACT: Artemisinin based combination therapy
*P. malariae*: *Plasmodium malariae*
*P. vivax*: *Plasmodium vivax*
*P. ovale*: *Plasmodium ovale*
*P. knowlesi*: *Plasmodium knowlesi*
aOR: Adjusted odds ratio
CI: Confidence interval
RR: Relative risk
CSP: Circumsporozoite
PCR: Polymerase chain reaction
CM: Cerebral malaria
PfEMP1: *Plasmodium falciparum* erythrocyte membrane protein 1
PIESP2: Parasite-infected erythrocyte specific protein 2
RDT: Rapid diagnostic test
IRS: Indoor residual spraying
ITNs: Insecticide-treated nets
SMC: Seasonal malaria chemo-prevention
PfSPZ: *Plasmodium falciparum* sporozoite
MDGs: Millennium Development Goals
ALMA: African Leaders Malaria Alliance
LLINs: Long-lasting insecticide treated nets
1,7-mRCTR: 1,7-malaria Reactive Community-based Testing and Response
